# Chip Protein U-Box Domain Truncation Affects Purkinje Neuron Morphology and Leads to Behavioral Changes in Zebrafish

**DOI:** 10.3389/fnmol.2021.723912

**Published:** 2021-09-24

**Authors:** Yasaman Pakdaman, Elsa Denker, Eirik Austad, William H. J. Norton, Hans O. Rolfsnes, Laurence A. Bindoff, Charalampos Tzoulis, Ingvild Aukrust, Per M. Knappskog, Stefan Johansson, Ståle Ellingsen

**Affiliations:** ^1^Department of Clinical Science, University of Bergen, Bergen, Norway; ^2^Department of Medical Genetics, Haukeland University Hospital, Bergen, Norway; ^3^Department of Biological Sciences, University of Bergen, Bergen, Norway; ^4^Department of Neuroscience, Psychology and Behavior, University of Leicester, Leicester, United Kingdom; ^5^Department of Biomedicine, Molecular Imaging Center, University of Bergen, Bergen, Norway; ^6^Department of Clinical Medicine, University of Bergen, Bergen, Norway; ^7^Department of Neurology, Neuro-SysMed Center of Excellence for Clinical Research in Neurological Diseases, Haukeland University Hospital, Bergen, Norway

**Keywords:** CHIP, *STUB1*, SCAR16, zebrafish, Purkinje cells, anxiety-like behavior

## Abstract

The ubiquitin ligase CHIP (C-terminus of Hsc70-interacting protein) is encoded by *STUB1* and promotes ubiquitination of misfolded and damaged proteins. CHIP deficiency has been linked to several diseases, and mutations in the human *STUB1* gene are associated with recessive and dominant forms of spinocerebellar ataxias (SCAR16/SCA48). Here, we examine the effects of impaired CHIP ubiquitin ligase activity in zebrafish (*Danio rerio*). We characterized the zebrafish *stub1* gene and Chip protein, and generated and characterized a zebrafish mutant causing truncation of the Chip functional U-box domain. Zebrafish *stub1* has a high degree of conservation with mammalian orthologs and was detected in a wide range of tissues in adult stages, with highest expression in brain, eggs, and testes. In the brain, *stub1* mRNA was predominantly detected in the cerebellum, including the Purkinje cell layer and granular layer. Recombinant wild-type zebrafish Chip showed ubiquitin ligase activity highly comparable to human CHIP, while the mutant Chip protein showed impaired ubiquitination of the Hsc70 substrate and Chip itself. In contrast to SCAR16/SCA48 patients, no gross cerebellar atrophy was evident in mutant fish, however, these fish displayed reduced numbers and sizes of Purkinje cell bodies and abnormal organization of Purkinje cell dendrites. Mutant fish also had decreased total 26S proteasome activity in the brain and showed behavioral changes. In conclusion, truncation of the Chip U-box domain leads to impaired ubiquitin ligase activity and behavioral and anatomical changes in zebrafish, illustrating the potential of zebrafish to study *STUB1*-mediated diseases.

## Introduction

C-terminus of Hsc70-interacting protein (CHIP), encoded by the *STUB1* (STIP1 homology and U-box containing protein 1) gene, is a dimeric co-chaperone that negatively regulates the efficiency of Hsp70 and Hsp90 chaperones by interfering with the ATPase cycles of the folding process (Ballinger et al., 1999). CHIP was the first ubiquitin ligase to be directly associated with molecular chaperones (McDonough and Patterson, 2003). The N-terminal TPR (tetratricopeptide repeat) domain is responsible for the interaction of CHIP with molecular chaperones, while the C-terminal U-box domain acts as an E3 ubiquitin ligase and facilitates ubiquitination of chaperone substrates for further proteasomal degradation (McDonough and Patterson, 2003). In addition, a central helical hairpin (HH) region together with another interacting surface in the U-box domain are involved in the dimerization and stability of CHIP (Zhang et al., 2005). CHIP is also known to have self-ubiquitination activity, which is independent of target ubiquitination and regulates CHIP ligase activity and turnover (Murata et al., 2001).

CHIP is ubiquitously expressed with highest levels in metabolically active tissues such as heart, brain, and skeletal muscles (Ballinger et al., 1999). Analysis of CHIP expression and localization in the rodent brain (Anderson et al., 2010; Shi et al., 2013) suggested co-localization of CHIP with the calcium-binding protein calbindin in cerebellar Purkinje cells of the cerebellum as the critical elements of precise motor coordination (Shi et al., 2013).

The dual activity of CHIP both in the ubiquitin-proteasome pathway and as a molecular co-chaperone have made CHIP a major player in the cellular protein quality control (PQC) system (Paul and Ghosh, 2015; Joshi et al., 2016). Studies on CHIP knockdown and knockout mouse models have shown that CHIP participates in the regulation of cellular senescence and mammalian longevity (Min et al., 2008). In addition, CHIP is involved in many signaling pathways and dysregulation of CHIP function has been implicated in several human diseases including cancers (Cheng et al., 2018; Tang et al., 2019), cardiac diseases (Naito et al., 2010; Cao et al., 2016), and other stress-related pathologies (Joshi et al., 2016). Moreover, mutations of CHIP causing inability to target protein substrates result in the formation of protein aggregates, and have been associated with the development of many neurodegenerative disorders including Alzheimer’s (Dickey et al., 2006), Parkinson’s (Tetzlaff et al., 2008), and polyglutamine diseases (Jana et al., 2005; Williams et al., 2009).

Several mutations in the *STUB1* gene have been reported in patients with recessive and dominant cerebellar ataxias (CA) (Ravel et al., 2021). CAs comprise a wide range of inherited and sporadic human neurodegenerative disorders in which progressive degeneration of the cerebellum results in poor balance and aberrant movement (Hersheson et al., 2012). So far, more than 30 *STUB1* mutations have been identified in patients with autosomal recessive spinocerebellar ataxia type 16 (SCAR16) (Stenson et al., 2017; Ravel et al., 2021). SCAR16 is characterized by early onset cerebellar ataxia, often associated with cognitive impairment and secondary infertility as a result of hypogonadotropic hypogonadism (De Michele et al., 2020). Very recently, heterozygous mutations in *STUB1* have been reported to cause dominant spinocerebellar ataxia type 48 (SCA48) (Ravel et al., 2021), which is characterized by a later age of onset and a wide range of variably associated phenotypes including parkinsonism, cognitive-psychiatric disorder, chorea, epilepsy, and endocrine dysfunctions (De Michele et al., 2020).

Insight into the pathological mechanisms associated with *STUB1* mutations is growing (Shi et al., 2013, 2014; Casarejos et al., 2014; Heimdal et al., 2014). We previously described the *in vitro* structural properties of selected *STUB1* recessive and dominant mutations, illustrating the substantial effects some of these mutations had on the stability and dimerization of CHIP (Pakdaman et al., 2017, 2021). However, molecular mechanisms and pathways affected by mutant CHIP proteins leading to human diseases remain largely unanswered.

In this study, we used zebrafish model to analyze the putative pathogenic role of the U-box domain in CHIP-mediated diseases. Mutations in the U-box domain have been reported for SCAR16/SCA48 patients and it is believed that dysfunction of the E3 ubiquitin ligase activity encoded by this domain is a key mechanism in the pathogenesis of these variants (Madrigal et al., 2019). For example, the SCAR16 variant, p.Thr246Met, was suggested to cause severe ataxia associated with cognitive deficiencies and hypogonadism in patients through loss of CHIP function to polyubiquitinate both chaperone-bound proteins and chaperone themselves (Shi et al., 2014). We first characterized the wild-type *STUB1* zebrafish ortholog (*stub1*) expression pattern and Chip protein function. Next, we generated the first zebrafish mutant for *stub1* by truncation of the Chip functional U-box domain by CRISPR/Cas9 technology. Wild-type zebrafish Chip showed ubiquitin ligase activity comparable to the mammalian orthologs in human and mouse, while homozygous mutants with truncated U-box domain had impaired ubiquitin ligase activity. Further, we showed that the lack of a functional U-box domain led to morphological changes in the Purkinje cell bodies and dendrites as well as reduced activity of 26S proteasome in the mutant brains. Mutant fish also displayed a significant decrease in body size and an altered pattern of explorative behavior associated with reduced anxiety.

## Materials and Methods

### Zebrafish Maintenance

Wild-type zebrafish (*Danio rerio*) with the TAB genetic background (cross between AB and Tübingen strains) were used. Zebrafish were maintained in the animal facility at the Department of Biological Sciences, University of Bergen, under standard conditions as described elsewhere (Aleström et al., 2019). In brief, fish were kept at 28.5°C in a 14 h/10 h light/dark cycle. All the experiments were conducted in compliance with the Norwegian Animal Welfare Act guidelines, Mattilsynet (FOTS application with FDF reference No. 17/119996). Fin clipping and body size measurements were performed on fish that were previously anesthetized in 164 mg/L Tricaine methanesulfonate solution.

### C-Terminus of Hsc70-Interacting Protein Structural Alignments

CHIP protein sequences for humans (*H. sapiens*), zebrafish (*D. rerio*), mouse (*M. musculus*), rat (*R. norvegicus*), chicken (*G. gallus*), frog (*X. tropicalis*), pufferfish (*T. rubripes*), and Japanese rice fish (*O. latipes*) were obtained from NCBI reference sequence database (O’Leary et al., 2016), and aligned using T-Coffee multiple sequence alignment tool (Notredame et al., 2000).

### Expression and Purification of Recombinant Zebrafish Chip Protein

Full-length *stub1* cDNA was amplified from wild-type and mutant zebrafish brain total cDNA, and the *Nco*I and *Not*I restriction sites were introduced by using gene-specific forward (5′-CCATGGAGAAGATGGCGAGCAGCCCAGAG-3′) and reverse (5′-GCGGCCGCGTAGTCCTCCACCCAG-3′) primers and Advantage PCR kit (Takara Bio Inc., Shiga, Japan). Resulting fragments were cloned into the pCRII-TOPO vector using the TOPO TA Cloning Kit (Thermo Fisher Scientific, MA, United States). Positive colonies were used for further sub-cloning into the prokaryotic expression vector pETM41 (EMBL, Heidelberg, Germany). The *Nco*I*/Not*I-digested *stub1* insert was ligated into the pETM41 vector using T4 DNA ligase (New England BioLabs, MA, United States). His-MBP-fusion Chip proteins were expressed in BL21-CodonPlus (DE3)-RP competent cells and purified as described previously (Pakdaman et al., 2017). The His-MBP-tagged proteins were stored in 100 mM HEPES, 100 mM NaCl, 5 mM DTT, and 10% glycerol.

### *In vitro* Ubiquitination Activity and Immunoblotting

*In vitro* ubiquitination reactions were performed on recombinant zebrafish and human CHIP as previously described (Pakdaman et al., 2017). Human CHIP proteins were purified from the bacterial expression vector pETM41, which was previously prepared by cloning CHIP full length cDNA from purchased vector pMXs.EXBi-STUB1-IRES-Puro (Cyagen Bioscience Inc., California) (Heimdal et al., 2014).

CHIP ubiquitination activity was tested for 2.5 μg of CHIP proteins during 1 h incubation at 37°C in the presence of 1 μg recombinant human His-HSPA8 (HSC71) (Lifespan Biosciences, WA, United States) and other components of ubiquitination reaction. Immunoblotting analyses were performed, using anti-HSC70/HSP73 (1:10,000, Cat. ADI-SPA-815, Enzo Life Sciences, Inc., NY, United States) and anti-CHIP antibody (1:2,000, Cat. C9243, Sigma-Aldrich, MO, United States) antibodies.

### RNA Extraction

A tissue panel containing heart, liver, skin, intestines, gonads, brain, thyroid, and head kidney of 2 years old zebrafish were subjected to RNA extraction by homogenization of frozen tissues in Trizol reagent using a Precellys 24 homogenizer (Bertin instruments, program: 5000-1 × 15-005). Phase separation was induced by adding chloroform, and the upper aqueous phase (RNA) was isolated by isopropanol. Additional RNA precipitation was performed in 0.1 × volume of 3 M sodium acetate, pH 5.2 and 2.5 × volume of 100% ethanol.

### Reverse Transcription and Droplet Digital PCR

First-strand cDNA was synthesized from 100 ng of total RNA using the Superscript III reverse transcriptase kit (Invitrogen, CA, United States). The ddPCR reaction was prepared in a mixture containing 2x QX200 ddPCR EvaGreen Supermix (Bio-Rad Laboratories, CA, United States), 0.25 μM forward (5′-GAGCTGCACGCCTATCTCAG-3′) and reverse (5′-CAGGTAATCGGGGATCTCGC-3′) primers, as well as 15 ng of cDNA templates. No-template control (NTC) and no-reverse transcriptase (NRT) reactions were included as negative controls in each run. Droplets of each sample were generated from 20 μl of reaction mixture and 70 μl of droplet generation oil (Bio-Rad) in the QX200 Automated Droplet Generator with DG8 cartridges (Bio-Rad). PCR amplification was carried out on 40 μl of generated sample droplets in heat-sealed 96-well plates using the C1000 thermal cycler (Bio-Rad) and the thermal profile (with ramping rate of 2°C/s): 95°C for 5 min, 40 cycles of denaturation at 95°C for 30 s and annealing at 60°C for 60 s, followed by 5 min cooling at 4°C, 1 cycle of 90°C for 5 min, and final hold at 4°C. Transcripts of *stub1* were quantified by using QuantaSoft analysis software (Bio-Rad) on the QX200 Droplet Reader (Bio-Rad) at a rate of 32 wells/h. Data were normalized to the negative controls and the number of specific copies per ng of initial cDNA was calculated for each sample. Graphical illustrations were prepared by GraphPad Prism version 8.4.2.

### cRNA Probe Synthesis

pExpress-1 vectors containing complete *stub1* cDNA sequence (ENSDART00000145075.3, ensemble genome database; Ensembl release 102—November 2020) was purchased (Source BioScience, Nottingham, United Kingdom) and verified by Sanger sequencing. Sense- and anti-sense cRNA probes were synthesized for 687 bp zebrafish *stub1* sequence (C70-C755) following plasmid linearization with *Not*I (New England BioLabs) and *Eco*RV (New England BioLabs) restriction enzymes, respectively, and purification of PCR-amplified *stub1* cDNA fragments containing SP6 (*Not*I-digested) and T7 (*Eco*RV-digested) promoter sites. PCR reactions were prepared by using forward and reverse primers for T7- (forward: 5′-CTGTTCCTCAGCCGCAAGTA-3′, reverse: 5′-TAATACGACTCACTATAGGGGTCAAAATGGCCAACACGC T-3′) and SP6- (forward: 5′-ATTTAGGTGACACTATAGAA CTGTTCCTCAGCCGCAAGTA-3′, reverse: 5′-GTCAAAAT GGCCAACACGCT-3′) containing fragments. Probe synthesis was performed using Digoxigenin (DIG) RNA labeling kit (Roche Molecular Biochemicals, Basel, Switzerland) and 400 ng of PCR product as template. Transcription reactions were terminated by addition of EDTA on ice, and cRNA probes were precipitated in a solution of 0.1 × volume of 4 M LiCl, 3.7 μg/μl tRNA (Roche), and 3 × volume of 100% ethanol.

### *In situ* Hybridization

TissueTek-embedded brains of adult zebrafish were cryosectioned (8 μm) on Superfrost Plus slides (Thermo Fisher Scientific) serially in the sagittal plane using a Leica CM3050S cryostat (Leica Biosystems, Wetzlar, Germany). *In situ* hybridization was performed as previously described (Sandbakken et al., 2012). Briefly, the sections were fixed in 4% paraformaldehyde and hybridized with *stub1* cRNA probes overnight at 65°C. For immunodetection, sections were incubated with sheep anti-DIG-AP antibody (1:2,000, Cat. 11093274910, Sigma-Aldrich) and color development was obtained during 30 min to 4 h incubation of sections in 4-nitro blue tetrazolium chloride and 5-bromo-4-chloro-3-indolyl phosphate-4-toluidine salt (Sigma-Aldrich) in the dark. After termination of the visualization reaction, the sections were mounted in 70% glycerol. *In situ* hybridization graphs were taken by Leica M420 microscope (Leica Microscope Systems), followed by digital processing.

### CRISPR/Cas9 Constructions

Potential target sites were identified within the *stub1* gene by using zifit^1^ and CHOP-CHOP^2^ webtools. Candidate sites were blasted against the whole genome of zebrafish^3,4^ and checked by using CHOP-CHOP and Integrated DNA Technology^5^ tools for the presence of off-targets. Single guide RNA (sgRNA) was prepared for the selected target site following instructions by Gagnon et al. (2014). In brief: *stub1* target site-specific oligo (5′-AGGTCAGAGGACTACGCGTGA-3′) was designed as a 60-base pair oligonucleotide containing an upstream T7 promoter (5′-TAATACGACTCACTATA-3′) and a downstream overlapping region (5′-GTTTTAGAGCTAGAAATAGCAAG-3′) which anneals to a constant oligonucleotide (5′-AAAAGCACCGACTCGGTGCCACTTTTTCAAGTTGATAAC GGACTAGCCTTATTTTAACTTGCTATTTCTAGCTCTAAAA C-3′) by mixing at the final concentrations of 20 μM, and during the PCR program: denaturation for 5 min at 95°C, decreasing temperature to 85°C by −2°C/s and then to 25°C by −0.1°C/s, and indefinite hold at 4°C. T4 DNA polymerase (300 units/ml, New England BioLabs) was used to fill in the annealed strands during 20 min incubation at 12°C. The reaction was terminated by adding the final concentration of 20 mM EDTA on ice followed by 20 min incubation at 75°C. DNA templates were further purified by using QIAquick PCR purification kit (Qiagen, Hilden, Germany), and products of the correct size were verified by DNA agarose gel electrophoresis. Transcription of sgRNAs were performed by using Amibon MEGAscript T7 Transcription Kit (Thermo Fisher Scientific).

### Microinjection and Development of Homozygous Mutant Fish

Zebrafish embryos were injected at the one-cell stage with 2–3 nl of a mixture containing 600 ng/μl Cas9 protein (PNA Bio Inc., CA, United States), 200 ng/μl sgRNA, and 300 mM KCl. Injection solutions were prepared on ice and incubated at 37°C prior to injection. Around 20 injected embryos (F0 generation) were collected at 48 h post fertilization (hpf) and screened for the presence of induced mutations around the target site (section “Genomic DNA Extraction and Genotyping”). The F0 generation were crossed with wild-type zebrafish (TL) at 3 months old. Male and female F1 fish with identical genotypes were crossed to generate the F2 homozygous mutants.

### Genomic DNA Extraction and Genotyping

Genomic DNA was extracted from individual zebrafish embryos and tail-cuts using standard protocols. Amplification of target sites was performed in a PCR reaction containing 0.2 μM forward (5′-TCTCCATTAATGAGCAGTTGGA-3′) and reverse (5′-ATGAAAGCGTCGATCACCTC-3′) primers, following the PCR program: 3 min of denaturation at 96°C, 35 cycles of amplification (96°C for 30 s, 55°C for 30 s, and 72°C for 30 s), final extension at 72°C for 10 min, and indefinite hold at 4°C. PCR products were further digested by 10 units/ml of *Mlu*I-HF restriction enzyme (New England BioLabs), and visualized on a 2.5% agarose gel.

### Targeted Gene Sequencing

A StrataClone PCR Cloning kit (Agilent, CA, United States) was used to clone PCR products of the target site into the pSC-A-amp/kan vector, and further transform the cloning products into *E. coli* competent cells (provided by the kit). Positive clones were identified by PCR analysis of selected colonies, using 0.5 μM T3 (5′-GCAATTAACCCTCACTAAAGGGA-3′) and T7 (5′-CCCTATAGTGAGTCGTATTA-3′) primer pair. PCR conditions were set up as follows: 10 min of denaturation at 94°C, 35 cycles of amplification (94°C for 30 s, 48°C for 30 s, and 72°C for 30 s), final extension at 72°C for 10 min, and indefinite hold at 4°C. PCR reactions were cleaned and sequenced by using illustra ExoProStar (Sigma-Aldrich) and 0.5 μM T3 primer. Sequencing was performed using an Applied Biosystems 3730 capillary sequencer (Applied Biosystems, CA, United States) at the Department of Medical Genetics, Haukeland University Hospital, Bergen, Norway. Results were analyzed by using SnapGene software (GSL Biotech LLC).^6^

### Zebrafish Magnetic Resonance Imaging

Magnetic resonance imaging (MRI) of zebrafish was performed on a 7T small animal MRI scanner (Bruker, MA, United States), with a 760 mT/m actively shielded imaging gradient insert. A 72 mm diameter transmit coil together with a phased array surface RF receive-only coil were used for excitation and detection of the MR signal. The system was connected to a Linux PC running Paravision 6.0.1 software (Bruker). Adult zebrafish were euthanized and placed in a 3D-printed plastic mold. High-resolution T2-weighted images were acquired by using TurboRARE (rapid acquisition with relaxation enhancement) spin-echo sequences with echo time 28.2 ms; repetition time 2,000 ms; RARE factor 8; slice thickness 0.2 mm; number of slices 12; field-of-view 8 × 4.5 mm; image matrix 200 × 112 pixels, resulting in an in-plane resolution of 40 um with 200 averages.

### 26S Proteasome Activity Assay

The 26S proteasome activity was measured as described previously by Kristiansen et al. (2007). In brief, zebrafish brain tissues were lysed and treated with 50 U/ml Benzonase Nuclease (Sigma-Aldrich) for 15 min on ice to digest nucleic acids. Ten micrograms of extracted protein were added to 100 μl proteasome reaction buffer containing 57 mM fluorogenic substrate SUC-LLVY-AMC (Sigma-Aldrich). Fluorescence was measured every minute for 60 min at 37°C using TECAN 96 well-plate reader (Tecan, Mannedorf, Switzerland) with the excitation/emission wavelengths of 360/440 nm. Non-specific substrate hydrolysis was measured by pre-incubation of proteins with 50 mM of the proteasome inhibitor Epoxomicin (Sigma-Aldrich) for 30 min at 37°C, and subtracted from each measurement. The corresponding corrected fluorescence measurements were plotted against time and the 26S proteasome activity was determined by calculating the slope of the regression line.

### Dot Blot Analysis

Soluble proteins were extracted from zebrafish brains in phosphate-buffered saline containing 100x Protease Inhibitor Cocktail (Sigma-Aldrich) under native conditions. Ten micrograms of protein were directly loaded onto a nitrocellulose membrane and blotted by A11 anti-oligomer antibody (Cat. AHB0052, Thermo Fisher Scientific). Recombinant wild-type and Thr246Met mutant human CHIP proteins (Pakdaman et al., 2017) were used as controls for negative and positive aggregation, respectively. Loading control samples were prepared by 10 min incubation of proteins at 72°C prior to loading, and subsequent blotting by anti-beta actin antibody (Cat. ab209869, Abcam, Cambridge, United Kingdom).

### Immunohistochemistry

For immunostaining, anti-parvalbumin (1:200, Millipore MAB1572), anti-Ataxin-3 (1:100, GeneTex GTX115032), anti-HSP70/73 (1:50, LSBio LS-B3700), and anti-Grin2a (NR2A) (1:100, Creative Diagnostics CPBT-66734RR) were used as the primary, and the anti-mouse Alexa Fluor Plus 555 (1:400, Cat. A32727, Thermo Fisher Scientific) and anti-rabbit Alexa Fluor Plus 488 (1:400, Thermo Fisher Scientific A32731) were used as secondary antibodies. Antibodies were diluted in PBST (PBS + 0.05% Triton X-100), 1x Casein Blocking Buffer (Sigma-Aldrich) before use. Cryosections (8 μm) were air-dried and washed with PBST before the primary antibody was added to the samples for overnight incubation at RT. The samples were then washed with PBST, and incubated in the secondary antibody for 45 min at RT. The slides were mounted using ProLong Glass medium containing NucBlue nucleic acid dye (Invitrogen). Analysis of cell death (TUNEL) was performed on cryosections by using the *In situ* Cell Death Detection Kit, Fluorescein (Roche).

Fluorescent images were obtained by TCS SP5 II confocal microscope (Leica Microsystems) and an Axio Scan.Z1 slide scanner (Carl Zeiss AG, Oberkochen, Germany). Images were processed using ImageJ (Schneider et al., 2012), and Alexa Fluor 555, Alexa Fluor 488, and NucBlue signals were pseudo-colored red, green and blue, respectively.

### Dendrite Orientation Analysis

NeuTube software (Feng et al., 2015) was used for neuronal tracing, using the anti-parvalbumin fluorescent signal on confocal-acquired z-stacks of the posterior cerebellum. Resulting 3-D rendered pictures were displayed using a perspective projection positioned at the origin of all axes, with neurons displayed as lines and selecting “direction” as a color mode. To quantity dendrite orientation, coded under that mode as a continuous gradient from green [0°–parallel to proximo-distal (P-D) axis] to red (90°–perpendicular to the P-D axis) through yellow (i.e. same amount of red and green, 45°), we generated RGB histograms of these pictures along their Y-(P-D) axis, using Image J. From these histograms, the difference between total red and green signals for each pixel row of the picture was calculated and plotted as absolute values, and on the resulting graphs, the total area of the predominantly green or red areas was measured to determine trends in dendrite orientation along the P-D axis.

### Morpho-Anatomical Quantification

To measure the average number of Purkinje cell bodies per individual, we analyzed wild-type and mutant zebrafish at 6 (wild-type: *n* = 3; mutant: *n* = 6) and 24 (wild-type: *n* = 3; mutant: *n* = 6) months of age. Parvalbumin-positive cell bodies were manually and blindly counted on three sagittal sections per individual, using the ImageJ Cell counter tool. The sections were imaged by a slide scanner in similar positions for each individual, covering adjacent and sequential positions along the medio-lateral axis. The average cross-section cell area was measured manually by ImageJ freehand drawing tool on three sagittal sections per individual. The number of Purkinje cell dendritic spines was quantified by using the LAS X software (Leica Microsystems). Spines along a 10 μm area of dendrites were manually counted on 9–10 dendrites in both wild type and mutants and the average of the dendritic spines per μm was calculated. Quantification of the dendrite diameter was performed by measuring the diameter at 9–10 random points along 10 μm of dendrites on 9–10 dendrites in both wild type and mutants. Dendritic spines were excluded in the measurement of the dendrite diameter, and the number of dendrites was chosen based on the visibility of dendrites.

### Behavioral Analysis

Behavioral experiments were conducted on adult zebrafish at 12 and 24 months of age in a dedicated zebrafish room with constant illumination and temperature (28°C). Behavioral recordings were captured using a digital camera (Point Gray Research Inc., BC, Canada), and FlyCapture software version 2.5.2.3 (FLIR Systems, OR, United States). EthoVision XT12 (Noldus Information Technology, Wageningen, Netherland) was used for video tracking. Quantification and analyses were performed blind to the genotype of the fish being examined.

For the novel tank diving test (Egan et al., 2009), individual fish were transferred into standard 3.5 L trapezoid tanks [27.9 × 22.5 × 11.5 × 15 cm, L (top) × L (bottom) × W × H], and their behavior was immediately recorded for 5 min. The amount of time spent in the bottom, middle, and top third of the tank, total distance swum, angular velocity, and latency to enter the top 25% zone of the novel tank were measured for each fish using EthoVision software. During the light/dark preference test, single fish were placed into a transparent plastic tank (30 × 20 × 12 cm, L × W × H) equally divided into black and white zones and with the water depth of 10 cm. After 5 min habituation, the swimming behavior of each fish was recorded from above for 5 min, evaluating the total amount of time spent in the white zone. The open field test was performed in an open tank (30 × 20 × 12 cm, L × W × H) filled with water to a depth of 10 cm. Following 5 min habituation, the total distance swum, the locomotor velocity, and the angular velocity were measured for each fish during 5 min by video tracking from above.

## Results

### Zebrafish Chip Is a Highly Conserved Homodimer With E3 Ubiquitin Ligase Activity

**FIGURE 1:**
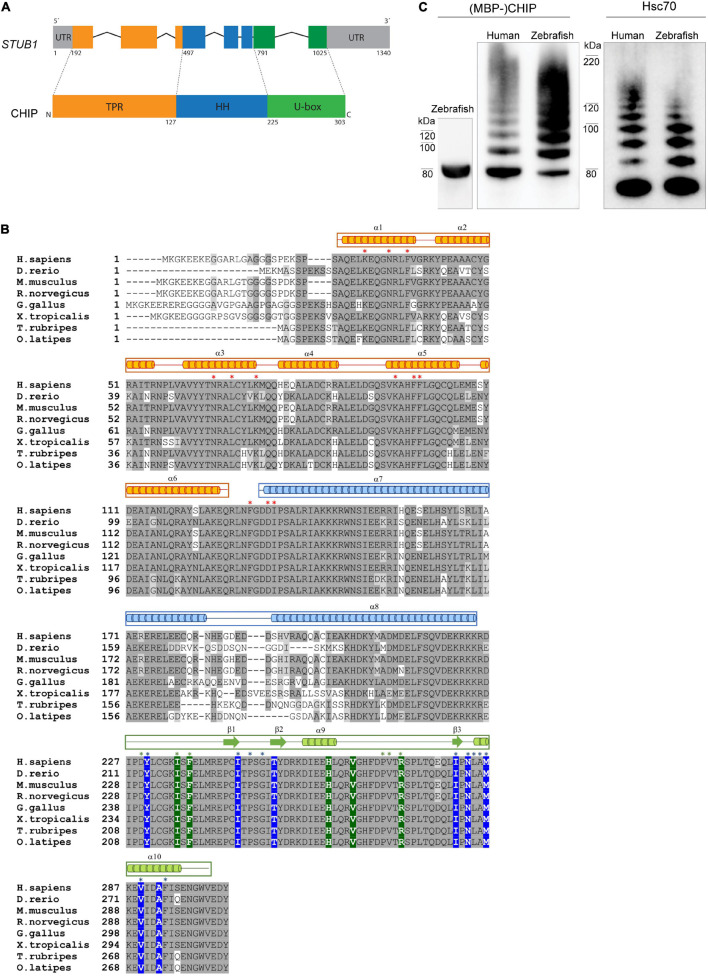
CHIP structure and ubiquitination activity is conserved among human and zebrafish. **(A)** Seven exons in the structure of *STUB1* gene correspond to the TPR, helical hairpin, and U-box domains of CHIP protein. **(B)** Alignment of CHIP sequence from human (*H. sapiens*), zebrafish (*D. rerio*), mouse (*M. musculus*), rat (*R. norvegicus*), chicken (*G. gallus*), frog (*X. tropicalis*), pufferfish (*T. rubripes*), and Japanese rice fish (*O. latipes*). The predicted position of secondary structures is displayed above the corresponding amino acid sequences in orange, blue, and green boxes corresponding to the TPR, helical hairpin, and U-box domains, respectively. Main residues involved in the U-box dimerization (in blue) as well as interactions via the TPR (in orange) and U-box (in green) domains are marked with background color (reported in zebrafish Chip) and/or asterisks (reported in mouse CHIP). This figure was produced by using T-Coffee (Notredame et al., 2000). **(C)** MBP-Chip recombinant protein was detected by Western blot analysis around 80 kDa (lane 1). Identical Hsc70- and self-ubiquitination activities were observed for human CHIP (lanes 2 and 4) and zebrafish Chip (lanes 3 and 5), shown by multiple ubiquitinated bands above MBP-CHIP (lanes 2 and 3) and Hsc70 (lanes 4 and 5).

We identified one copy of the *stub1* zebrafish gene in the zebrafish genome based on searches in Ensembl^7^ and Uniprot^8^ databases, thus our analysis refers to the gene annotated as “*stub1”* in the Ensembl genome database (ENSDARG00000045228, Ensembl release 102—November 2020). The primary structure of the CHIP protein (Figure 1A) includes three domains: the N-terminal TPR, the central helical hairpin (HH), and the C-terminal U-box. A structure-based protein sequence alignment between human, monkey, mouse, rat, chicken, frog, zebrafish and additional fish orthologs (Figure 1B) shows that all three domains are clearly present in all represented species. The primary amino acid sequence identity of CHIP between zebrafish and human is high (79%). The reported key residues for the interaction of CHIP with Hsp70/Hsp90 molecular chaperones and E2 enzymes (Zhang et al., 2005; Xu et al., 2006) were found to be identical between zebrafish, mouse, and other species (Figure 1B, marked with green background/asterisk for E2 and red asterisk for chaperones). Likewise, the main amino acids located at the core of U-box interfaces in the beta sheet structures and helix 10 (Zhang et al., 2005; Xu et al., 2006) are identical across all species (Figure 1B, marked with blue background/asterisk), while the sequence of the helical dimerization segment in the helix 7 and 8 varies considerably between species (Figure 1B).

To further investigate the functional conservation of CHIP between human and zebrafish, we generated recombinant full-length MBP-tagged zebrafish Chip protein (Figure 1C, lane 1) and tested its ability to ubiquitinate itself and the Hsc70 chaperone (Figure 1C, lanes 2–5). Western blotting using anti-CHIP and anti-HSC70 antibodies revealed full ubiquitination activities for zebrafish Chip (Figure 1C, lane 3 and 5) that were highly comparable to the human CHIP control (Figure 1C, lane 2 and 4), demonstrated by a series of high molecular bands representing ubiquitinated MBP-CHIP and Hsc70.

### *stub1* RNA Is Widely Expressed in Zebrafish

**FIGURE 2:**
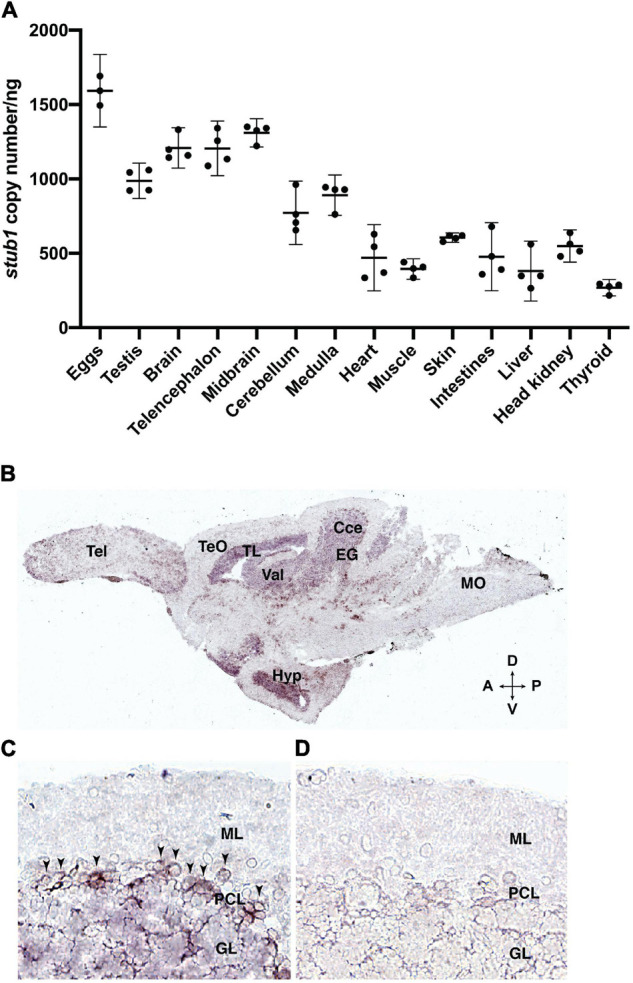
*stub1* mRNAs are expressed in different tissues and in the Purkinje cells of adult zebrafish cerebellum. **(A)** Scatter plot of ddPCR data presenting the absolute number of *stub1* transcripts per ng of cDNA for different zebrafish tissues. Each dot represents a biological replicate. Means and standard deviations are indicated by the lines and bars in the graph (*n* = 4). **(B)** DIG-labeled *stub1* cDNA probes were synthesized, and used for visualization of *stub1* mRNA expression in adult zebrafish brain sections, using *in situ* hybridization assay. **(C)** Detailed view of cerebellum where *stub1* positive cells in the Purkinje cell layer are indicated by arrowheads. **(D)** Negative control corresponding to the same area as shown in **(B)**. Tel, telencephalon; TeO, tectum opticum; TL, torus longitudinalis; Val, valvula cerebelli; Cce, Corpus cerebelli; EG, eminentia granularis; Mo, medulla oblongata; Hyp, hypothalamus; A, anterior; P, posterior; D, dorsal; V, ventral; ML, molecular layer; PCL, Purkinje cell layer; GL, granular layer.

To investigate the expression pattern of *stub1* in zebrafish and compare it to mammals, we studied diverse tissues using ddPCR. Variable levels of *stub1* transcript were detected in all tissues examined (Figure 2A and Supplementary Tables 1, 2), with the highest level in the eggs, testis, and brain, and the lowest levels in thyroid.

### *stub1* mRNA Is Present in the Purkinje Cell Layer of the Zebrafish Cerebellum

The localization of *stub1* mRNA in the brain of adult zebrafish was investigated by *in situ* hybridization, using DIG-incorporated cRNA probes. The *stub1* anti-sense probe-hybridized sections demonstrated signals in the cerebellar corpus (Cce) and eminentia granularis (EG), including cells in the Purkinje cell layer (PCL) and the granular layer (GL) (Figures 2B,C). We also observed staining in valvula cerebelli (Val) and torus longitudinalis (TL) (Figure 2B). Outside of the cerebellum and midbrain area, we detected notable expression in hypothalamus (Hyp) (Figure 2B). Negative controls hybridized with the *stub1* sense probes showed no staining (Figure 2D). These results indicate the expression of *stub1* mRNA in the zebrafish cerebellum, most likely in the Purkinje cell bodies and granular cells.

### Generation of Mutant Zebrafish With Truncated Chip U-Box Domain

**FIGURE 3:**
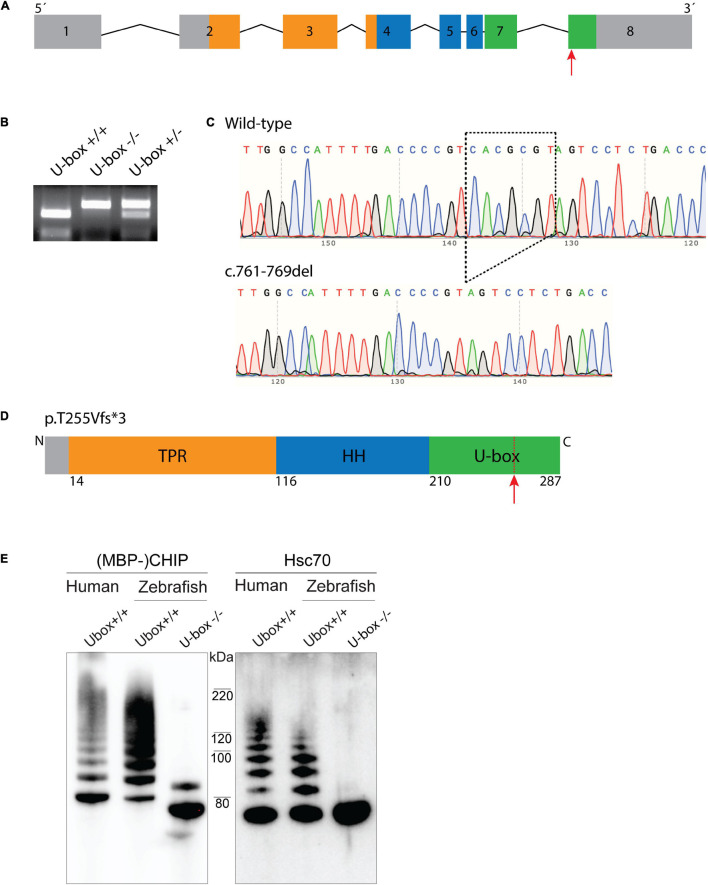
Zebrafish U-box^− /−^ expressing truncated Chip with impaired ubiquitin ligase activity were generated by CRISPR/Cas9. Zebrafish *stub1* gene was targeted for mutagenesis at the last exon **(A)**, leading to an introduction of 7 bp-deletion mutation in this exon (c.761-769del) which was detected by *Mlu*I restriction digestion **(B)** and DNA sequencing **(C)**. The 761-769del mutation is predicted to result in a frameshift and early stop codon and truncation of C-terminal U-box domain **(D)**. Recombinant Chip proteins were produced from the cDNA of U-box^− /−^ and wild-type zebrafish, and further examined for *in vitro* ubiquitin ligase activity on Chip (**E**, lane 1–3) and Hsc70 substrates (**E**, lane 4–6). Compared to the wild-type human CHIP (**E**, lane 1 and 4) and wild-type zebrafish Chip (**E**, lane 2 and 5) controls, U-box truncated zebrafish Chips showed impaired self-ubiquitination (**E**, lane 3) and Hsc70 ubiquitination activities (**E**, lane 6).

Mutant fish encoding truncated Chip functional U-box domain (U-box^–/–^) were generated by CRISPR/Cas9 technique. The sgRNA was designed to target the last exon (exon 8) of the *stub1* gene (Figure 3A), which encodes the C terminal U-box domain of Chip (Figure 3D). An F0 adult fish with the highest frequency of mutations in its F1 offspring (77%) was selected as founder for establishing a homozygous mutant line. After outcrossing the founder fish, heterozygous F1 fish with a 7 bp-deletion in the *stub1* target site were identified by *Mlu*I restriction digestion and DNA sequencing (Figures 3B,C), and crossed to breed F2 homozygous U-box^–/–^ mutants. The 7 nucleotide-deletion mutation encodes a frameshift mutation at Threonine 255 and an early stop codon after encoding three amino acids [p.Thr255Valfs^∗^3, removing the 28 most C-terminal residues of the U-box domain (Figure 3D)].

Homozygous U-box^–/–^ zebrafish displayed normal early development, longevity, and breeding abilities. Homozygous fish were raised and phenotypically characterized at 6, 12, and 24 months after fertilization.

### U-Box^–/–^ Fish Express Truncated Chip Protein With Impaired Ubiquitin Ligase Activity

To investigate the effects of the U-box domain truncation on Chip E3 enzymatic activity in zebrafish, *stub1* cDNA was amplified from wild-type and U-box^–/–^ brains, and sub-cloned into the pETM41 bacterial expression vector. Recombinant MBP-tagged wild-type and mutant Chip proteins were further purified and subjected to *in vitro* ubiquitin ligase activity assay (Figure 3E). Western blotting demonstrated similar ubiquitination activities for the human CHIP control (Figure 3E, lane 1 and 4) and wild-type zebrafish Chip (Figure 3E, lane 2 and 5) as observed by multiple bands with increasing molecular weight. In the mutant zebrafish Chip, we observed a mono self-ubiquitination (Figure 3E, lane 3) and complete lack of activity on Hsc70 substrate (Figure 3E, lane 6), demonstrated by the lack of bands with increased molecular weight. In addition, a small molecular weight shift was observed for the U-box^–/–^zebrafish MBP-Chip protein (Figure 3E, lane 3), confirming the amino acid truncation induced by the Thr255Valfs^∗^3 mutation in this protein.

### U-Box^–/–^ Fish Show Progressive Reduction of Size Compared to Wild-Type Siblings

**FIGURE 4:**
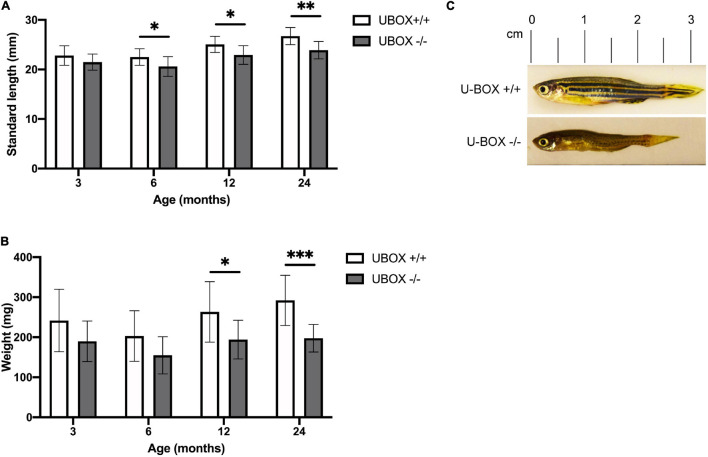
U-box^− /−^ fish are associated with smaller body size compared to their wild-type siblings. Quantification of standard length **(A)** and body weight **(B)** are presented for wild-type and U-box^− /−^ zebrafish in indicated age groups (*n* = 10). Data shown are means and error bars indicate standard deviation. Statistically significant differences are shown by asterisks (^∗^*p* < 0.05; ^∗∗^*p* < 0.01; ^∗∗∗^*p* < 0.001, Student’s *t*-test). **(C)** Representative images of U-box^− /−^ and wild-type zebrafish (female) at 24 months of age.

In order to study the progressive effect of mutant Chip on fish growth, we measured the standard length and body weight of wild-type and U-box^–/–^ fish (*n* = 10) at 3, 6, 12, and 24-month timepoints (Figure 4 and Table 1). The measurements were performed blind to the fish genotypes, and the *stub1* mutation status for each fish was identified afterward. At 3 months of age, no significant differences were detected between wild-type and mutant fish. At 6 months, mutant fish showed a slower growth in standard length compared to the wild-type (*t*-test, *p* = 0.03). Significant differences in length were also observed at 12 months (*t*-test, *p* = 0.01) and 24 months (*t*-test, *p* = 0.001), and mutant fish at 24 months old were on average 2.8 mm shorter than their wild-type siblings (Figures 4A,C). The weight at 3 and 6 months were not significantly different, but a trend of decreased weight could be observed for the mutant. However, the mutant fish showed significantly reduced weight at 12 (*t*-test, *p* = 0.02) and 24 months (*t*-test, *p* = 0.0005), with an average weight of 94.5 mg (*t*-test, *p* = 0.0005) lower than their wild-type siblings at 24 months (Figure 4B). The measurements at each state were performed on the same number of male and female fish of each genotype to avoid a bias toward gender on the body size differences observed across the two groups.

**TABLE 1:** The standard length (mm) and body weight (mg) of wild-type and U-box^–/–^ fish at indicated time points.

**Age (month)**		**3**	**6**	**12**	**24**
Standard length (mm)	U-box ^+/+^	22.81 ± 1.9	22.5 ± 1.6	25.06 ± 1.6	26.7 ± 1.7
	U-box ^–/–^	21.48 ± 1.62	20.6 ± 1.9	22.9 ± 1.8	23.9 ± 1.7
Weight (mg)	U-box ^+/+^	241.7 ± 77.9	202.8 ± 63.1	263.3 ± 75.4	292.1 ± 62.6
	U-box ^–/–^	189.9 ± 50.6	154.9 ± 46.5	194.2 ± 48.3	197.6 ± 34.3

*Data shown are means ± standard deviations.*

### Magnetic Resonance Imaging Reveals Intact Brain Structures in the U-Box^–/–^ Fish

**FIGURE 5:**
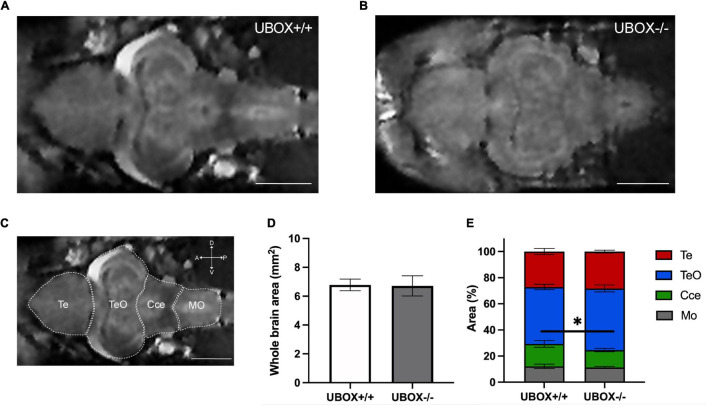
Magnetic resonance imaging of wild-type and U-box^− /−^ zebrafish brains demonstrate similar intact structures. MRI of the adult zebrafish brain at 24 months revealed no significant alterations in the overall brain structures between wild-type **(A)** and mutant **(B)** zebrafish. **(C)** Different regions of a wild-type zebrafish brain are indicated on a representative MRI image. Section area of the whole brain **(D,E)** the relative area of different regions **(D)** were calculated by using Fiji (ImageJ) software (31). Data shown are means and error bars indicate standard deviations (^∗^*p* < 0.05, Student’s *t*-test). Scale bars in **(A–C)**: 1 mm. Te, telencephalon; TeO, tectum opticum; Cce, corpus cerebelli; Mo, medulla oblongata.

MRI of the brain was performed on adult zebrafish at 24 months of age (*n* = 4). No major differences were observed for the general structure or morphology of the brain regions between wild-type and mutant fish (Figures 5A–C). Measurement of whole brain area revealed no differences between mutant and wild-type fish (Figure 5D), however, the area corresponding to the cerebellum was slightly smaller in the mutant fish (Figure 5E, *t*-test, *p* = 0.03746).

### Chip U-Box Truncation Causes a Disturbed Growth Pattern and Morphology in Purkinje Cell Dendrites

**FIGURE 6:**
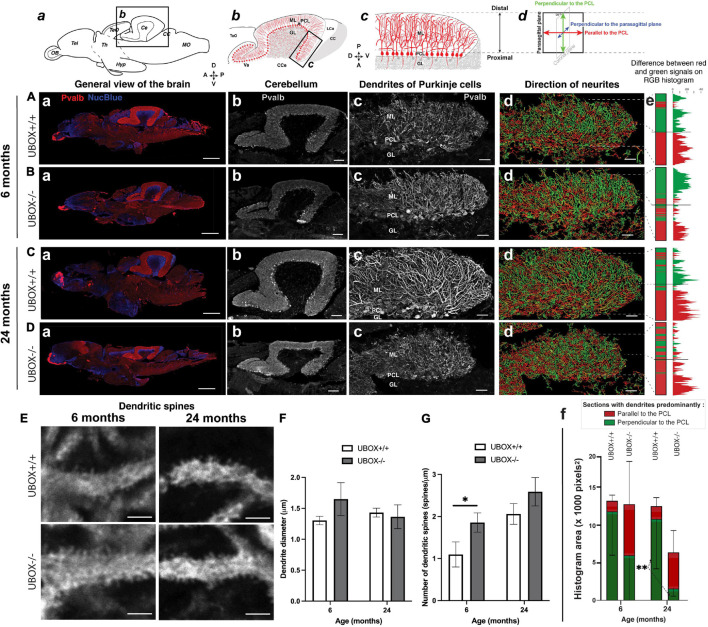
U-box^− /−^ mutants present a progressive dendrite arbor disorganization and an initial increase in dendritic spines. **(A–E)** Fluorescent immunohistochemistry on 8 μm sagittal cryosections of wild-type (**A,C,** and top **E**) and mutant (**B,D**, and bottom **E)** fish using Parvalbumin as a marker for Purkinje cells. The diagrams on top of **(a–d)** columns show the localization, orientation and structure of the underlying pictures. **(A–Da)** are slide scanner acquisitions showing the general anatomy of the brain, and **(A–Db)** are close-ups of the cerebellum from the same pictures. **(A–Dc)** are maximum z-projections of confocal stacks centered on the molecular layer of the posterior cerebellum, and **(A–Dd)** are the 3D renderings of neuronal tracing analysis on the stacks presented in c. The adjacent color bars on the right **(e)** highlight trends in the dendrite orientation along the proximal-distal axis of the molecular layer, each quantified as the histogram area for the distal part of the molecular layer and indicated time points **(f)**. **(E)** pictures are single confocal sections presenting a zoomed view of dendritic spines in the median to distal areas of the molecular layer. **(F,G)** present quantification results of the dendrite diameter **(F)** and the number of dendritic spines **(G)** at the two time-points as means ± standard deviations (^∗^*p* < 0.05 and ^∗∗^*p* < 0.01, Student’s *t*-test). Scale bars in **(A–D)**: 400 μm in a, 100 μm in b and 30 μm in **(c–d)**. Scale bars in **(E)**: 2,5 μm.

We investigated the structure and organization of Purkinje cells in the cerebellum of wild-type and mutant zebrafish at 6 (wild-type: *n* = 3; mutant: *n* = 6) and 24 (wild-type: *n* = 3; mutant: *n* = 6) months using immunohistochemistry of sagittal cryosections incubated with anti-parvalbumin (Pvalb) antibody (Bae et al., 2009). We tested a panel of anti-CHIP antibodies, both commercially available and custom made (Supplementary Table 3), but could not identify any antibody with high specificity for zebrafish Chip. Pvalb-positive structures were detected in several parts of the brain at 6 and 24 months with the strongest expression observed in the cerebellum of both wild-type and U-box^–/–^ fish (Figures 6Aa–Da). The overall size of the cerebellum, as shown by the crown of Pvalb-positive dendrites, was found to be smaller in mutant fish at 24 months (Figures 6Bb,Db) compared to the wild-type control (Figures 6Ab,Cb). However, the ratio between the cerebellum and other brain structures (cerebellum + telencephalon) was unchanged (Supplementary Figure 1A). The thickness of the molecular layer was also smaller in the mutant brains at 24 months, yet it was proportional to the size of the cerebellum (Supplementary Figure 1B) between wild-type and mutant brains, indicating no clear change in relative size. Altogether, we did not observe gross signs of cerebellar atrophy in the mutants up to 24 months.

In the zebrafish cerebellum, the somata of the Purkinje cells are located in a single-celled layer. Each cell soma extends primary dendrites into the molecular layer (ML) in a stereotypic pattern following a single para-sagittal (translobular) plane and arborizing into flat, fan-shaped structure with multiple branches (Figure 6c). In the wild-type, we observed the dendrites as long, coherent neurites visible in each section (Figures 6Ac,Cc). In the posterior part of the cerebellum of mutants, the dendrites appeared generally more disorganized, and highly segmented dendrites were observed in each section, indicating that their growth was less strictly ordered compared to the observation in the wild-type fish. The apparent disorganization of the Purkinje cell dendrites was observed in the mutant brains at both examined time points (Figures 6Bc,Dc).

To closer analyze the observed disturbances of the Purkinje cell dendrites in mutants, we performed neuronal tracing on the confocal images in the posterior part of cerebellum (Figure 6d). In this analysis, the dendrites are displayed as lines with different colors in the sagittal sections, with green indicating growth along the proximal-distal (P-D) axis (relative and perpendicular to the PCL) while red indicates growth parallel to the PCL and perpendicular to the P-D axis. The color gradient goes from parallel to the P-D axis (100% green, 0°) to perpendicular to the P-D axis (100% red, 90°). At 45°, the amount of red and green were counted as equal. In the wild-type, dendrites were mostly orientated along the P-D axis (green) in the distal half of the cerebellum ML, and showed more branching away from the P-D axis (red) in the proximal half of the ML (Figures 6Ad,Cd). This is visualized with the color bar oriented along the proximal-distal axis of the posterior part of the sagittal cerebellum sections (Figure 6e). For the wild-type, the color of the bar is largely split in two blocks, the proximal (red) and the distal (green) block, reflecting the strongest branching proximal to the Purkinje cell bodies (Figures 6Ae,Ce). In the mutant fish, we generally observed a shift from growth along the P-D axis (green) toward growth perpendicular to the P-D axis (red) in the distal part of the ML (Figures 6Bd,Dd). We could quantify this as a reduction of the total number of distal green dendrites (Figures 6Be,De). This pattern was observed as a trend in 6 months old mutant fish, while at 24 months a statistically significant reduction was observed in the total number of distal filaments along the P-D axis in the mutant compared to the wild-type fish (*t*-test, *p* = 0.0093, Figure 6f). Overall, these findings indicate a progressive disruption in the spatial organization of Purkinje cell dendrites in U-box^–/–^ fish with an increasing alternate pattern of orientation in the ML.

Next, we investigated the detailed complexity of Purkinje cell dendrites (wild-type: *n* = 3; mutant: *n* = 3) by using different morphometric parameters. We observed similar dendrite diameter between genotypes (Figures 6E,F and Table 2), indicating no significant swelling or shrinking of the dendrites although standard deviation was higher in data from mutants compared to the controls suggesting greater variability. We also noticed a trend toward an increased number of spines on the U-box^–/–^ dendrites compared to the wild-type at both ages examined (Figures 6E,G and Table 2), although this increase only appeared to be statistically significant at the 6-month time point (*t*-test, *p* = 0.024).

**TABLE 2:** Morphological properties of Purkinje cells in wild-type and U-box^–/–^ fish at indicated time points.

**Age (month)**		**6**	**24**
Dendrite diameter (μm)	U-box^+/+^	1.3 ± 0.04	1.43 ± 0.04
	U-box^–/–^	1.65 ± 0.03	1.36 ± 0.11
Number of dendritic spines (Spines/μm)	U-box^+/+^	1.1 ± 0.28	2.06 ± 0.14
	U-box^–/–^	1.86 ± 0.13	2.59 ± 0.19
Number of cell bodies	U-box^+/+^	272 ± 35	314 ± 22
	U-box^–/–^	199 ± 30	223 ± 44
Cross sectional cell body area (μm^2^)	U-box^+/+^	51.73 ± 20.8	63.55 ± 23.6
	U-box^–/–^	54.25 ± 22.32	45.43 ± 20.95

*Data shown are means ± standard deviations.*

### Purkinje Cell Bodies of U-Box^–/–^ Fish Are Smaller and Fewer in Number

**FIGURE 7:**
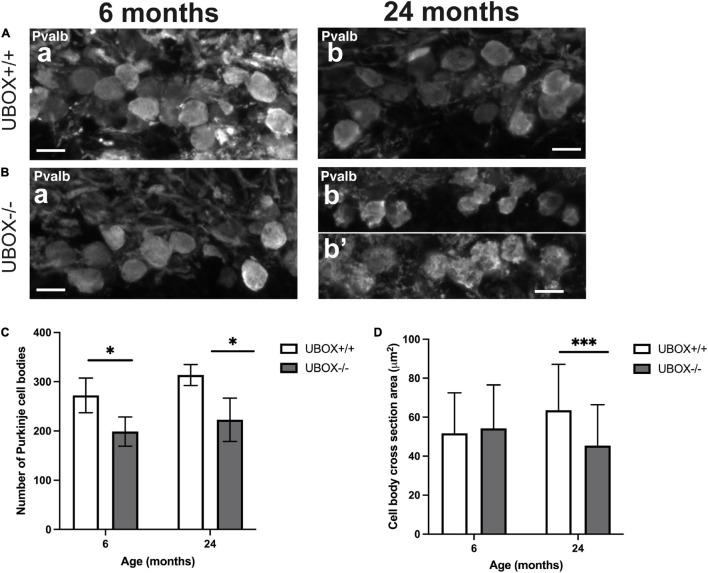
The number and size of Purkinje cell bodies are progressively reduced in U-box^− /−^ fish. Fluorescent immunohistochemistry on 8 μm sagittal cryosections on wild-type **(A)** and mutant **(B)** at 6 **(a)** and 24 **(b,b’)** months fish using Parvalbumin as a marker for Purkinje cells. **(C,D)** present quantification results for the average number of Purkinje cell bodies **(C)** and average cell body cross-section area **(D)** at the two time-points as means ± standard deviations. Statistically significant differences are shown by asterisks (^∗^*p* < 0.05 and ^∗∗∗^*p* < 0.001 Student’s *t*-test). Scale bars in **(A,B)**: 10 μm.

Quantification of the Purkinje cell bodies in wild-type and U-box^–/–^ cerebellum showed a significant loss in the mutants at 6 and 24 months of age. The number of cell bodies decreased by 26.83% at 6 months (*t*-test, *p* = 0.012) and by 28.98% at 24 months (*t*-test, *p* = 0.013) in the U-box^–/–^ fish (Figures 7Aa,Ab,Ba,Bb,C and Table 2). We also measured the size of Purkinje cell bodies in wild-type and mutant fish. No significant differences could be observed at 6 months (Figures 7Aa,Ba,D and Table 2). However, at 24 months we observed irregular shaped cell bodies and a 31.6% reduction in cell body area (*t*-test, *p* = 1.6e-14) for the mutant Purkinje cells (*n* = 382) compared to the wild-types (*n* = 116) (Figures 7Ab,Bb,D and Table 2).

### U-Box^–/–^ Fish Exhibit Defects in Protein Quality Control

**FIGURE 8:**
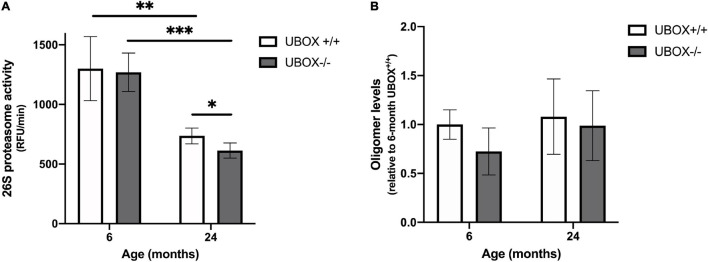
Reduced 26S proteasome activity was detected in U-box^− /−^ fish. **(A)** Chymotrypsin-like activity was measured in brain tissues of U-box^− /−^ and wild-type fish at 6 and 24 months (*n* = 4). Statistically significant differences are shown by asterisks (^∗^*p* < 0.05; ^∗∗^*p* < 0.01; ^∗∗∗^*p* < 0.001, Student’s *t*-test). **(B)** Oligomer expression quantified from dot blot analysis in brain tissues of U-box^− /−^ and wild-type fish at 6 and 24 months (*n* = 3). Data shown are means and error bars indicate standard deviations.

We investigated the effect of Chip functional deficiency on 26S proteasome activity and protein oligomerization in zebrafish brains (*n* = 4) at 6 and 24 months of age. Total activity of the 26S proteasome declined at 24 months compared to 6 months in both wild-type (*t*-test, *p* = 0.006) and mutant fish (*t*-test, *p* = 0.0002, Figure 8A) groups, which is in line with the previously reported age-dependent decrease in proteasome activity of human and other vertebrates (Dasuri et al., 2009; Kelmer Sacramento et al., 2020). No differences were evident in the proteasome activity of U-box^–/–^ compared to the wild-type fish brains at a 6-month time point. However, a significant reduction was demonstrated in 26S proteasome activity of 24 months old U-box^–/–^ brains compared to their wild-type siblings (*t*-test, *p* = 0.036, Figure 8A).

We assessed protein oligomerization in U-box^–/–^ fish as another indicator of a damaged protein quality control system, using a recently developed anti-oligomer (A11) antibody that recognizes oligomers of Aß, polyQ, prion, and insulin (Kayed et al., 2003). Dot blot analysis using the A11 antibody showed no significant difference in oligomer abundance in U-box^–/–^ brain lysates compared to wild-type controls at 6 and 24 months (Figure 8B and Supplementary Figure 2). In addition, examination of protein expression for other substrates of CHIP including the glutamate-binding subunit of N-methyl-D aspartate Receptor, NR2A, Hsp70 and ataxin-3 by immunohistochemistry of wild-type (*n* = 3) and mutant (*n* = 6) zebrafish brain sections at 24 months did not reveal any changes in response to the truncation of U-box domain in Chip (Supplementary Figure 3). Taken together, these results suggest a negative effect on cellular proteasome activity, caused by the U-box truncation of the mutant Chip enzyme. However, these effects did not result in an apparent accumulation of specific oligomers nor differences in the expression of three known CHIP protein substrates.

### Chip U-Box Truncation in Mutant Fish Is Associated With Altered Behavior Correlated to Less Anxiety

**FIGURE 9:**
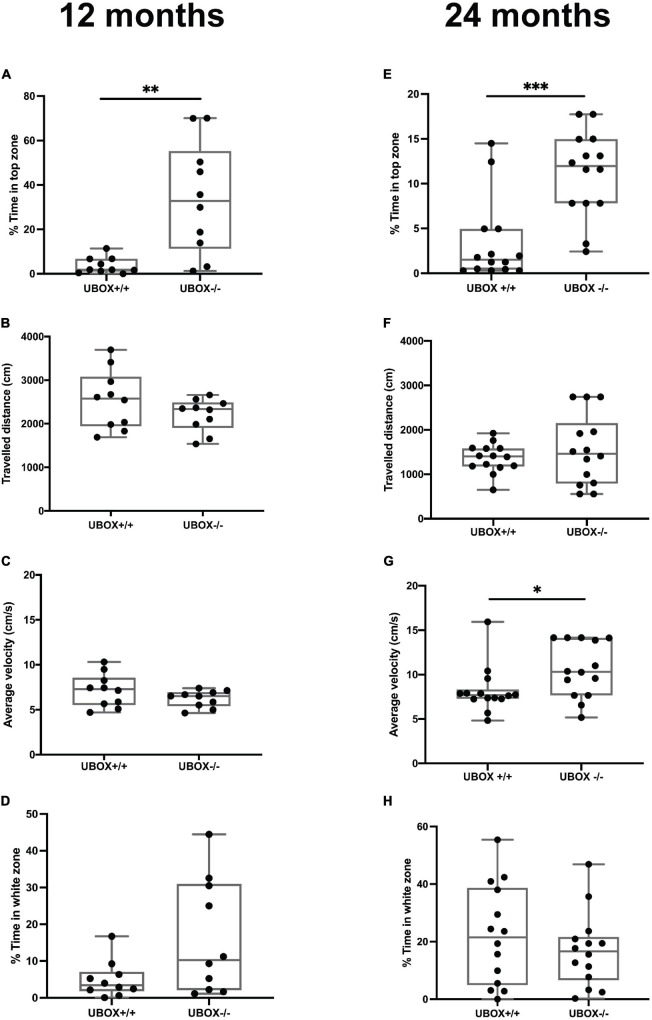
U-box^− /−^ fish exhibit less anxiety-like behavior compared to the wild-type fish. The anxiety-like behavior of wild-type and U-box^− /−^ fish was measured by novel tank diving and light/dark preference tests. In the novel tank diving test, the fraction of time spent in the top third zone of the novel tank **(A,E)**, the distance traveled **(B,F)**, and average velocity **(C,G)** was quantified and compared for each genotype group (*n* = 10–14 per group) at 12 **(A–C)** and 24 months **(E–G)**. In the light/dark preference test, the fraction of time spent in the white zone of the tank was quantified and compared for each group (*n* = 10–14 per group) at 12 **(D)** and 24 **(H)** months. Data shown are the lower and upper quartiles (Q1–Q3) and the whiskers show range of values outside Q1-Q3. Statistically significant differences are shown by asterisks (^∗^*p* < 0.05; ^∗∗^*p* < 0.01; ^∗∗∗^*p* < 0.001, Student’s *t*-test).

Behavioral changes related to anxiety were investigated for the wild-type and U-box^–/–^ fish at 12 (*n* = 10) and 24 (*n* = 14) months of age using the novel tank diving and light/dark preference tests (Figure 9). In the novel tank diving test, homozygous mutant fish spent significantly more time in the top third of the tank compared to their wild-type siblings at 12 (*t*-test: *p* = 0.0007, Figure 9A) and 24 (*t*-test: *p* = 0.0002, Figure 9E) months of age, indicating reduced anxiety-like behavior in U-box^–/–^ fish. We also observed a significant increase in the average swimming velocity in the 24 months old U-box^–/–^ fish compared to the wild-type controls (*t*-test: *p* = 0.0374, Figure 9G). Further analysis of these data showed no significant differences in latency to enter the top 25% of the novel tank for the wild-type and mutant fish at 12 and 24 months (Supplementary Figure 4). This indicates that these fish explore the tank and enter the top zone in a similar manner, although the mutant fish tend to stay longer at the top of the tank. There were also no significant differences between the fish in traveled distance in the novel tank (Figures 9B,F) and the amount of time spent in the white zone in light/dark preference test (Figures 9D,H). We further investigated locomotion behavior in the open field test, where the fish of different genotypes showed similar activities in terms of total traveled distance, average velocity, and angular velocity at 12 and 24 months of age (Supplementary Figure 5).

## Discussion

Mutations in the *STUB1* gene cause recessive spinocerebellar ataxia type 16 (SCAR16) and dominant spinocerebellar ataxia type 48 (SCA48) in humans (Ravel et al., 2021). Alterations in the CHIP U-box domain has previously been reported to cause severe biochemical defects as well as additional clinical manifestations for the SCAR16 variants including Thr246Met (Heimdal et al., 2014; Shi et al., 2014; Pakdaman et al., 2017) and Met240Thr (Madrigal et al., 2019). Therefore, the U-box domain seems to play a significant role in the pathogenesis of CHIP, and in the present study, we aimed to characterize the effects of truncation of this domain in zebrafish. Using CRISPR/Cas9 technique, we generated a mutation in the zebrafish *stub1* that caused a truncation of the Chip U-box domain. Characterization of the homozygous mutant zebrafish revealed impaired Chip ubiquitin ligase activity, associated with reduced body size, disturbances of cerebellar Purkinje cells and behavioral changes. Phenotypes were observed mainly at adult stages, while mutant fish showed apparently normal development during embryo, larval and juvenile stages. These data indicate that loss of the Chip U-box domain might not interfere with early development, possibly due to the presence of other proteins and mechanisms that are able to compensate for the loss of normal Chip function enabling relatively normal growth and cellular development. Observations reported in human SCA48 patients with CHIP-mediated ataxias (De Michele et al., 2020) point to adult-onset phenotypes, while mice models of CHIP deficiency were reported to mainly show normal early development (Dai et al., 2003). It is likely, that with time, the impaired protein quality control system leads to the gradual loss of neurons, a hallmark of the progressive nature of several neurodegenerative diseases, and thus primarily later phenotype manifestationsin our mutant fish.

Sequence alignment showed the presence of identical amino acids in the CHIP binding sites for chaperones and E2 ubiquitin conjugating enzymes as well as a similar dimerization interface at the U-box domain across mouse and zebrafish orthologs, based on the position of functional residues previously identified in crystal structures of mouse and zebrafish CHIP (Zhang et al., 2005; Xu et al., 2006). We showed that zebrafish Chip has ubiquitin ligase activity on Chip and Hsc70 substrates similar to the human ortholog. This strongly indicates the presence of conserved interactions with the E2 conjugating enzyme through the C-terminal (U-box domain) as well as with the chaperones via its N-terminal (TPR domain) to mediate the transfer of ubiquitin both to itself and to the substrates. It could be speculated that the apparent lower ubiquitination activity toward the Hsc70 substrate for the zebrafish Chip compared to human CHIP (Figures 1C, 2E) indicates potential differences in their catalytic activity resulting from variations in the amino acid sequence of the N-terminal substrate-binding domain. However, further studies of the CHIP binding properties of the two orthologs are needed before firm conclusions can be drawn.

A previous study in humans indicated high *STUB1 m*RNA expression in skeletal muscle and heart, and to a lesser extent in pancreas and brain (Ballinger et al., 1999). In contrast, we observed the highest levels of *stub1* in the zebrafish adult brain as well as eggs and testes. The high amount of *stub1* transcripts in the eggs and testes is interesting in light of the previously reported association with hypogonadism and reproductive impairments in some patients with SCAR16 as well as mouse models (Shi et al., 2014). Our findings indicate telencephalon (containing cerebral cortex and the homolog of the mammalian hippocampus) and midbrain, cerebellum and hypothalamus as parts of the zebrafish brain with the most *stub1* transcripts. These results are consistent with data from mouse and rat showing the highest *Stub1* expression in the cerebellum, pons, medulla, hippocampus, and cerebral cortex (Anderson et al., 2010; Shi et al., 2014), and also with data provided by the Human Protein Atlas, where RNA expression levels of *STUB1* are reported to be higher in cerebral cortex, hippocampus, and midbrain compared to cerebellum and spinal cord (Uhlén et al., 2015).^9^ Our *in situ* hybridization results on brain sections revealed *stub1* mRNAs in several parts of the cerebellum of adult zebrafish, including Purkinje cell layer. This is consistent with previous studies in humans and mice reporting wide expression of *STUB1* in the molecular and granular layers of cerebellum with high abundance in Purkinje cells (Anderson et al., 2010; Shi et al., 2014).

We further studied the functional significance of Chip in zebrafish by using a stable and viable mutant homozygous line, containing a CRISPR/Cas9-induced deletion mutation causing a truncation of the functional U-box domain of Chip. The induced truncation removed critical amino acids located at the core of the U-box interface in beta sheet 3 and alpha-helix 10 (Figure 1B), and completely disrupted Chip ubiquitin ligase activity toward the Hsc70 substrate. In case of self-ubiquitination activity, we observed a band corresponding to mono ubiquitination for mutant Chip proteins. While the origin of this observation remains unknown, it could suggest that non-specific interactions between the E2 enzyme and Chip is sufficient to mediate the addition of one ubiquitin molecule to the Chip, while this interaction is not strong enough to facilitate non-covalent bindings between ubiquitin molecules and the subsequent formation of polyubiquitin chains (Soss et al., 2011).

Immunohistochemistry of mutant brain sections with anti-Pvalb antibody revealed significant alterations in the total number and morphology of Purkinje cells, including Purkinje cell dendrite morphology and growth patterns. Decreased numbers of Purkinje cells were previously reported in the cerebellum of compound heterozygous SCAR16 patients with the U-box truncating mutation Glu238^∗^ and the Met211Ile substitution (Bettencourt et al., 2015). Similar findings were also reported in mouse animal models, where CHIP deficiency resulted in Purkinje cell loss and an increased number of pyknotic nuclei as well as severe dendritic swelling, mimicking phenotypes of human patients with disordered ubiquitination (Shi et al., 2014). There is a high degree of conservation reported for the cytoarchitecture and connectivity of cerebellar neurons between mammals and teleost fish (Hashimoto and Hibi, 2012). Cerebellar Purkinje neurons are one of the key neuron types in the cerebellum with characteristic planar fan-shaped dendrites extending into the ML. These dendrites are arranged to fill spaces with little overlap, a configuration that has been found to be essential for formation of numerous synapses with more than 100,000 parallel fibers and with each climbing fiber (Fujishima et al., 2018). Our observed changes to dendritic growth patterns and morphology in the mutants indicate a disturbance in their stereotypic organization, growth and branching. We also observed trends of increased number of dendritic spines and dendritic swelling in the mutant fish. Such changes can possibly have a significant impact on the relative amounts of excitatory inputs to the Purkinje neurons, and consequently the Purkinje cell outputs to the vestibular nucleus. In addition, biogenesis and homeostatic plasticity of dendritic synapses are known to be regulated by ubiquitin pathways (Mabb and Ehlers, 2010). Changes in synaptic activity result in ubiquitin-dependent regulation of synaptic proteins, which in turn regulate downstream pathways implicated in learning and memory (Mabb and Ehlers, 2010). Therefore, future studies are essential to elucidate whether neurotransmission pathways are affected by the disorganized dendrites and to investigate the potential effect of impaired ubiquitination on the overall cerebellar function and altered behavior in these mutant fish. The mutant fish displayed morphological changes in Purkinje cells that are partially overlapping with the human phenotype for SCAR16/SCA48. In addition, MRI data indicate slight differences in their cerebellum size compared to the wild-types suggesting initial atrophy of the cerebellum, a hallmark of the human phenotype. We could not confirm cell death phenotype during analysis of cerebellum sections at this stage (Supplementary Figure 6). However, based on the progressive nature of the disease, we cannot exclude the possibility of a progressive cerebellar atrophy at later stages than included in our analysis.

The anti-CHIP antibody we have used in this study could sufficiently detect purified, recombinant zebrafish Chip during immunoblotting assays (Figures 1C, 3E). However, despite extensive testing of a number of commercial and custom-made antibodies, we did not manage to identify or generate an antibody that could specifically detect zebrafish Chip during immunohistochemistry of brain sections. Thus, we were not able to directly study the putative differences in the Chip protein localization of the U-box^–/–^ mutants compared to the wild-type Chip in the zebrafish brain.

Interaction of CHIP with different chaperones and a broad range of substrates makes CHIP a master regulator of various protein networks and cellular pathways. Studies have shown the importance of CHIP in maintaining homeostasis for a large group of proteins associated with cell signaling, development, neurodegeneration, and cancer (Joshi et al., 2016). CHIP has also been implicated in the regulation of cellular senescence as CHIP knockout mice show decreased longevity and lower total body weight (Min et al., 2008). Reduced proteasome activity and elevated levels of toxic oligomers were also reported in these mice. Similarly, we have observed a significant decrease in the body size of mutant zebrafish at the age of 12 and 24 months. Also, the total activity for the 26S proteasome was reduced in mutant zebrafish at 24 months although this reduction was not associated with accumulation of oligomeric proteins such as tau oligomers in brain tissues. To further elucidate possible effects of the loss of ubiquitination activity in Chip, we examined if the protein levels of some of known CHIP substrates including the NMDA receptor NR2A, Hsp70 and ataxin-3 had been altered. Our immunohistochemistry analyses did not show significant alterations of these Chip substrates in mutant brain sections (Supplementary Figure 3). We therefore hypothesize that the lack of U-box domain function in the U-box^–/–^ fish can possibly be compensated for by the TPR domain, which can eliminate some substrates through other degradation pathways thereby maintaining normal levels of these substrates in the mutant fish. Lysosomal degradation of α-synuclein by the TPR domain of CHIP has previously been reported (Shin et al., 2005), and the TPR domain has also been shown to participate in proteasomal degradation of NR2A via direct interactions with F-box ubiquitin ligases (Nelson et al., 2006). Further investigations into the protein levels of additional Chip substrates in the U-box^–/–^ mutants, as well as null-mutants for Chip, can improve our understanding of the selective degradation pathways for each substrate mediated by specific domains of CHIP.

The U-box^–/–^ zebrafish spent significantly more time in the top zone of the novel tank, suggesting reduced anxiety-like behavior and aberrant exploration patterns in a novel environment. Consistent with the high levels of *stub1* expression detected in the hypothalamus of the zebrafish brain (Figure 2), such behavior could possibly reflect changes in the hypothalamus as previously described (Wei et al., 2020). Similarly, mice deficient for CHIP have been reported with increased amount of time spent in the open arms of the elevated plus maze, as well as spatial learning errors in the Barnes maze, which could suggest hippocampal damage and cerebellar dysfunction (Shi et al., 2014). In contrast, mammalian models of SCAR16 harboring a CHIP-Thr246Met mutation were reported to have increased anxiety, together with age-dependent sensorimotor deficiencies and social and cognitive deficits (Shi et al., 2018). *In vitro* findings showed that CHIP-Thr246Met retained other co-chaperoning functions despite being impaired for E3 ubiquitin ligase activity. Phenotypic differences observed for the CHIP-Thr246Met mice compared to the loss-of-function CHIP mice support the hypothesis that disease-causing mutations do not simply reflect total loss of CHIP activity. Similarly, the Chip proteins expressed by homozygous mutant zebrafish models in the present study have a truncated and non-functional U-box domain, yet are intact for the TPR and central helical hairpin domains. Therefore, these animals might present phenotypes different from those induced by complete loss of Chip proteins. The recent finding that *STUB1* mutations also can cause the dominant spinocerebellar ataxia disease, SCA48, emphasizes that the underlying mechanisms of *STUB1*-mediated ataxias remain poorly understood. Future studies on Chip zebrafish models with total loss-of-function, knock-in pathogenic mutations and potential dominant negative mutations will be important to improve our understanding of potential phenotypic differences caused by different types of Chip deficiency.

In conclusion, our findings illustrate a conserved pattern of CHIP expression and enzymatic function for human and zebrafish orthologs. The phenotypes described for the mutant zebrafish with truncated U-box domain are partially overlapping with the phenotypes reported in SCAR16/SCA48 human patients as well as mammalian models of CHIP deficiency. These data could open new doors for the potential use of zebrafish in modeling *STUB1*-related cerebellar ataxias.

## Data Availability Statement

The original contributions presented in the study are included in the article/Supplementary Material, further inquiries can be directed to the corresponding author/s.

## Ethics Statement

The animal study was reviewed and approved by the Norwegian Food Safety Authority.

## Author Contributions

YP, SE, WN, LB, CT, IA, PK, and SJ conceived the project and planned the experiments. YP, EA, WN, HR, ED, and SE performed experiments and prepared figures and tables. All authors analyzed and discussed the results, wrote the manuscript, read, and approved the final manuscript.

## Conflict of Interest

The authors declare that the research was conducted in the absence of any commercial or financial relationships that could be construed as a potential conflict of interest.

## Publisher’s Note

All claims expressed in this article are solely those of the authors and do not necessarily represent those of their affiliated organizations, or those of the publisher, the editors and the reviewers. Any product that may be evaluated in this article, or claim that may be made by its manufacturer, is not guaranteed or endorsed by the publisher.

## Footnotes

1
zifit.partners.org/ZiFiT

2
chopchop.cbu.uib.no

3
ensembl.org

4
NCBI.nlm.nih.gov

5
idtdna.com

6
snapgene.com

7
ensembl.org/zebrafish

8
uniport.org

9
proteinatlas.org
