# Supplementary material for: Chip Protein U-Box Domain Truncation Affects Purkinje Neuron Morphology and Leads to Behavioral Changes in Zebrafish
Source: Front Mol Neurosci. 2021 Sep 24;14:723912. doi: 10.3389/fnmol.2021.723912 (PMC8497888; doi:10.3389/fnmol.2021.723912)
Supplement: Supplementary file 1 [file Data_Sheet_1.PDF]

Supplementary material

Table S1. Measured *stub1* copy number by ddPCR for four biological replicates of zebrafish tissues.

	***stub1* copy number/ng cDNA**				
	**R1**	**R2**	**R3**	**R4**	**Mean ± SD**
**Eggs**	1494.93	1592.53	1691.2		1592.89±98.13
**Testis**	1044.27	921.6	924.27	1060.27	987.6±74.96
**Brain**	1157.87	1197.87	1331.2	1144.53	1207.87±85.29
**Telencephalon**	1133.87	1256.53	1088.53	1341.87	1205.2±115.49
**Midbrain**	1221.87	1349.87	1325.87	1341.87	1309.87±59.51
**Cerebellum**	962.67	762.67	656	706.67	772±134.37
**Medulla**	762.67	928	928	944	890.67±85.66
**Heart**	544	336	370.67	629.33	470±139.86
**Muscle**	408	440	397.33	336	395.33±43.51
**Skin**	577.6	620.27	620.27	601.6	604.93±143.91
**Intestines**	358.93	678.93	478.93	390.93	476.93±143.91
**Liver**	348.27	561.6	265.6	348.27	380.93±126.59
**Head kidney**	480	514.67	637.33	562.67	548.67±68.14
**Thyroid**	293.33	217.07	288	277.33	268.93±35.21

Table S2. Calculated p-values and levels of statistical significance between the average *stub1* copy number of zebrafish tissues. Statistically significant differences are shown by asterisks (*, p<0.05; **, p<0.01; ***, p<0.001, Student's *t* test).

	**1**	**2**	**3**	**4**	**5**	**6**	**7**	**8**	**9**	**10**	**11**	**12**	**13**
**1 (Eggs)**													
**2 (Testis)**	<0.0001***												
**3 (Brain)**	0.0004***	0.1065											
**4 (Telencephalon)**	0.0003***	0.1163	>0.9999										
**5 (Midbrain)**	0.0232*	0.0018**	0.9596	0.9511									
**6 (Cerebellum)**	<0.0001***	0.1241	<0.0001***	<0.0001***	<0.0001***								
**7 (Medulla)**	<0.0001***	0.9727	0.0023**	0.0025**	<0.0001***	0.8860							
**8 (Heart)**	<0.0001***	<0.0001***	<0.0001***	<0.0001***	<0.0001***	0.0044**	<0.0001***						
**9 (Muscle)**	<0.0001***	<0.0001***	<0.0001***	<0.0001***	<0.0001***	0.0001***	<0.0001***	0.9973					
**10 (Skin)**	<0.0001***	0.0001***	<0.0001***	<0.0001***	<0.0001***	0.4580	0.0088**	0.7664	0.1501				
**11 (Intestines)**	<0.0001***	<0.0001***	<0.0001***	<0.0001***	<0.0001***	0.0059**	<0.0001***	0.9999	0.9937	0.8226			
**12 (Liver)**	<0.0001***	<0.0001***	<0.0001***	<0.0001***	<0.0001***	0.0001***	<0.0001***	0.9864	>0.9999	0.094	0.9747		
**13 (Head kidney)**	<0.0001***	<0.0001***	<0.0001***	<0.0001***	<0.0001***	0.0961	0.0007***	0.9955	0.5926	0.9999	0.9982	0.4517	
**14 (Thyroid)**	<0.0001***	<0.0001***	<0.0001***	<0.0001***	<0.0001***	<0.0001***	<0.0001***	0.1943	0.8346	0.001***	0.1578	0.9214	0.0113*

Table S3. Anti-CHIP antibodies evaluated as unspecific against zebrafish Chip.

Antibody	Epitop	Host	Company	Catalog No.
**Anti-STUB1**	aa 218-267	Rabbit	Sigma-Aldrich	SAB210328
**Anti-STUB1, N-terminal**	aa 1-50	Rabbit	Sigma-Aldrich	SAB2103799
**Anti-CHIP, C-terminal**	aa 251-268	Rabbit	Sigma-Aldrich	C9243
**STUB1 polyclonal**	aa 236-263	Rabbit	Thermo Fisher Scientific	PA5-35110
**Polyclonal anti-human STUB1**	aa 236-263	Rabbit	Lifespan Biosciences	LS-C241389
**IHC-plus polyclonal anti-human STUB1**	aa 219-231	Goat	Lifespan Biosciences	LS-B6443
**STUB1 antibody, N-terminal**	aa 1-50	Rabbit	Aviva System Biology	ARP 43058
**STUB1 antibody**	aa 291-303	Goat	Aviva System Biology	OALA05653
**CHIP/STUB1 antibody**		Rabbit	Novus International	NBP1-30936
**Anti-zebrafish Chip antibody**	aa 160-180	Rabbit	Custom-made (available upon request)	
	aa 200-216	Rabbit		

Abbreviations: aa, amino acid.

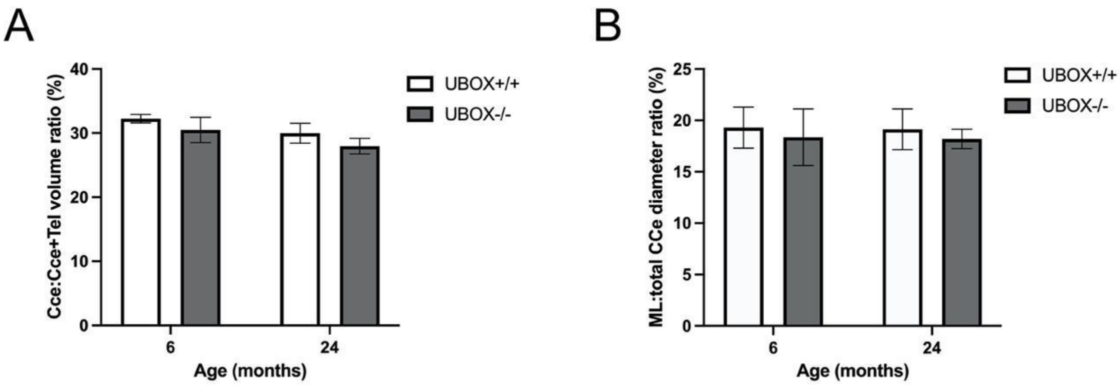

Figure S1. The size of the cerebellum relative to other brain parts was estimated to be similar between wild-type and U-box$^{-/-}$ fish. The total volume of cerebellum relative to the total volume of cerebellum and telencephalon (A), and the thickness of cerebellum molecular layer at the posterior part relative to the diameter of the total cerebellum (B) were measured by ImageJ and plotted for each genotype group at indicated time points. Data shown are means and error bars indicate standard deviations.

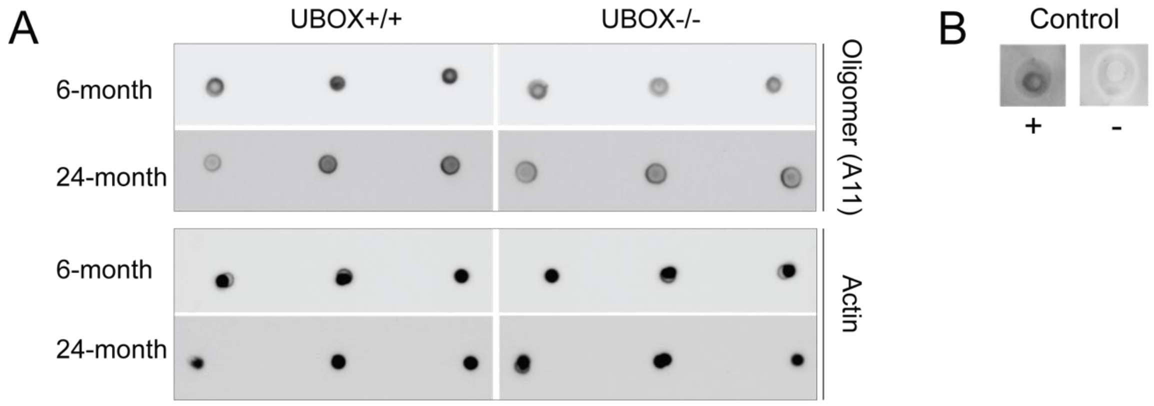

Figure S2. Protein accumulations were not detected in the U-box$^{-/-}$ brains. A) Dot blot analysis of oligomer levels in brain tissues of U-box$^{-/-}$ and wild-type fish at 6 and 24 months, using A11 anti-oligomer antibody. B) Positive and negative oligomer expression controls shown by dot blot analysis.

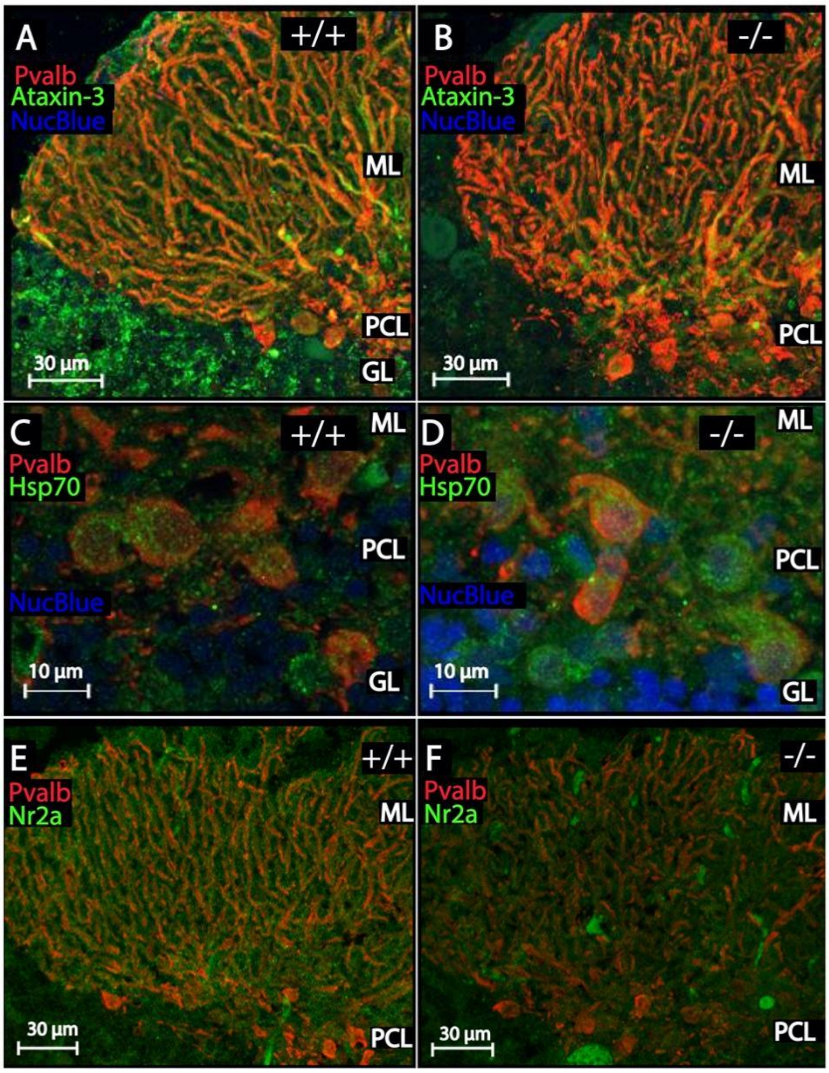

Figure S3. CHIP substrates show undisturbed expression patterns in mutant compared to wild-type zebrafish cerebellum. Immunofluorescent staining on sagittal cryosections of adult zebrafish brains, imaged using a confocal microscope. Green staining display Ataxin-3 (A and B), Hsp 70 (C and D), and Nr2a (E-F) expression in the wild-type (A, C, E) and mutant (B, D, F) sections. All stainings are overlaid with Pvalb (in red) staining. The blue staining corresponds to NucBlue-dyed nuclei. ML, molecular layer; PCL, Purkinje cell layer; GL, granular layer.

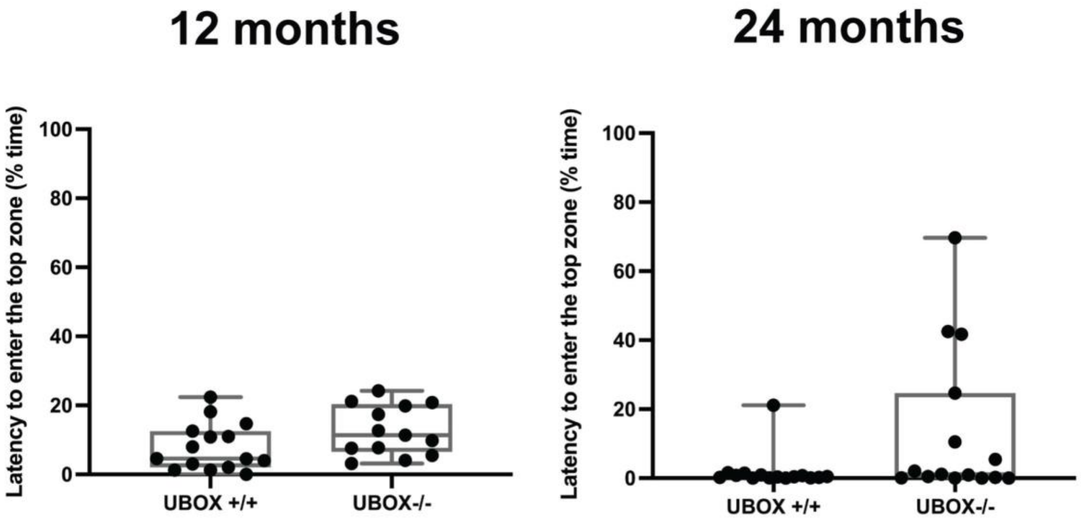

Figure S4. U-box–/– and wild-type fish showed similar patterns for entering the top zone of the novel tank. The latency to enter the top 25% of the tank was measured during the novel tank diving assay. Data are plotted for indicated genotypes against fraction of time as lower and upper quartiles (Q1-Q3) and the whiskers show range of values outside Q1-Q3.

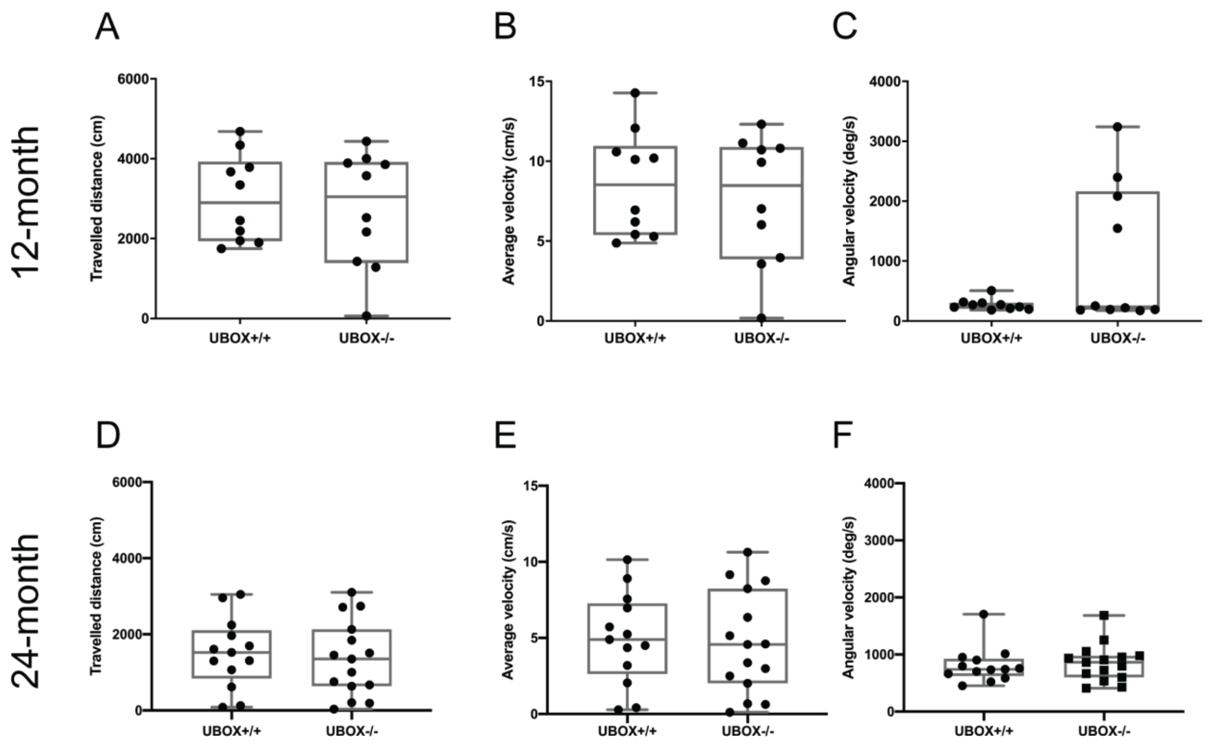

Figure S5. U-box$^{-/-}$ and wild-type fish exhibited similar locomotion behavior. The locomotion behavior of wild-type and U-box$^{-/-}$ fish was measured in open field test by quantification of the total travelled distance (A, D), the average velocity (B and E), and the angular velocity (C and F) for each indicated genotype group (n=10-14 per group) at 12 (A-C) and 24 (D-F) months. Data shown are the lower and upper quartiles (Q1-Q3) and the whiskers show range of values outside Q1-Q3.

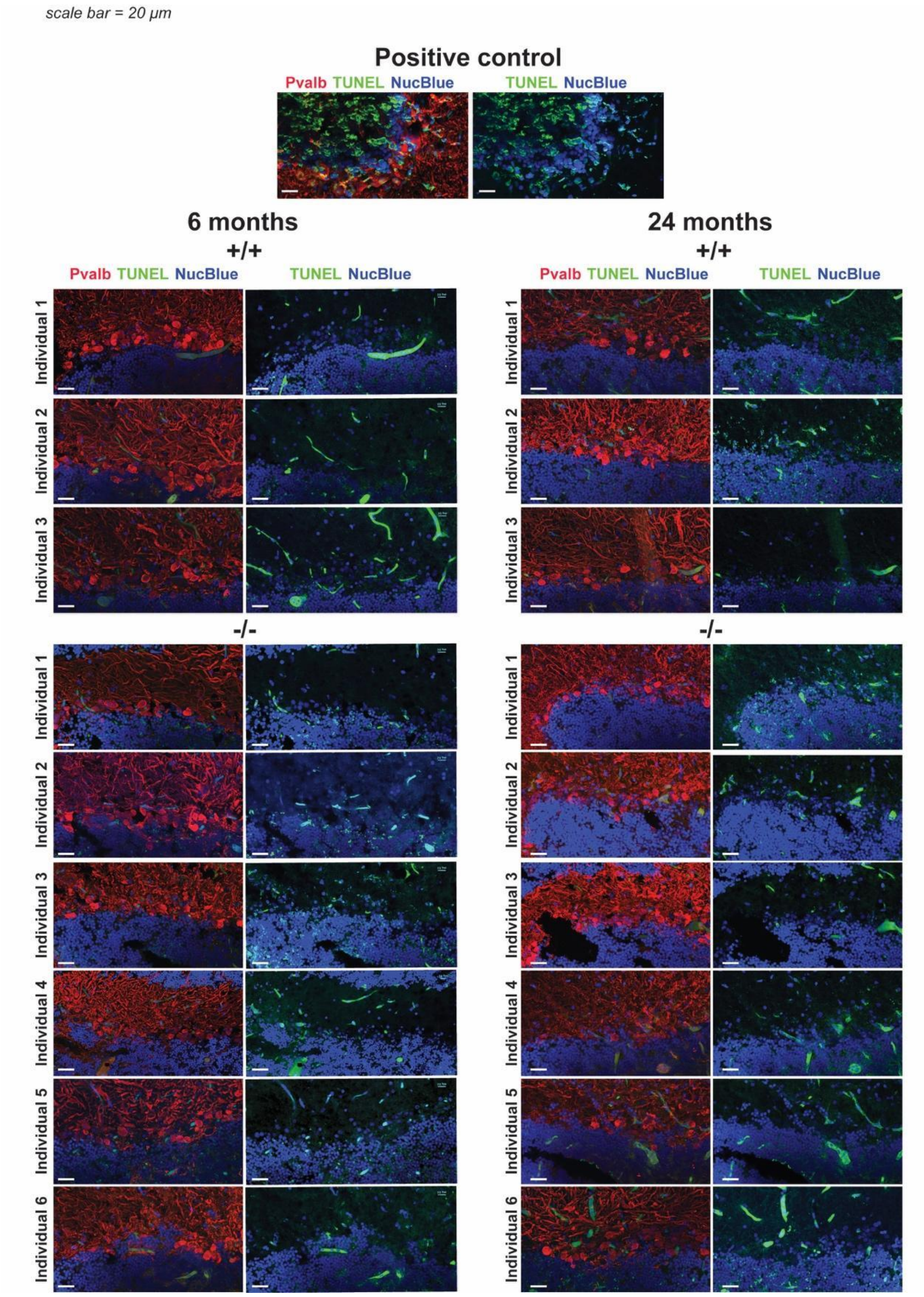

Figure S6. Cell death analysis of brain sections in adult zebrafish. Fluorescence TUNEL (in green) labelling of brain cryosections for wild-type and mutant zebrafish at 6 and 24 months of age. All stainings are overlaid with Pvalb (in red) staining. The blue staining corresponds to NucBlue-dyed nuclei. Positive controls were prepared by DNaseI treatment prior to labeling.
